# Naturally Occurring Genetic Alterations in Proximal TCR Signaling and Implications for Cancer Immunotherapy

**DOI:** 10.3389/fimmu.2021.658611

**Published:** 2021-05-03

**Authors:** Andrew Kent, Natalie V. Longino, Allison Christians, Eduardo Davila

**Affiliations:** ^1^ Division of Medical Oncology, Department of Medicine, University of Colorado, Aurora, CO, United States; ^2^ Human Immunology and Immunotherapy Initiative, University of Colorado, Aurora, CO, United States; ^3^ University of Colorado Comprehensive Cancer Center, Aurora, CO, United States; ^4^ Department of Medicine, University of Colorado, Aurora, CO, United States

**Keywords:** T cell (antigen) receptor, cellular immunotherapy, cytokines, T cell signaling, cellular engineering

## Abstract

T cell-based immunotherapies including genetically engineered T cells, adoptive transfer of tumor-infiltrating lymphocytes, and immune checkpoint blockade highlight the impressive anti-tumor effects of T cells. These successes have provided new hope to many cancer patients with otherwise poor prognoses. However, only a fraction of patients demonstrates durable responses to these forms of therapies and many develop significant immune-mediated toxicity. These heterogeneous clinical responses suggest that underlying nuances in T cell genetics, phenotypes, and activation states likely modulate the therapeutic impact of these approaches. To better characterize known genetic variations that may impact T cell function, we 1) review the function of early T cell receptor-specific signaling mediators, 2) offer a synopsis of known mutations and genetic alterations within the associated molecules, 3) discuss the link between these mutations and human disease and 4) review therapeutic strategies under development or in clinical testing that target each of these molecules for enhancing anti-tumor T cell activity. Finally, we discuss novel engineering approaches that could be designed based on our understanding of the function of these molecules in health and disease.

## Introduction

**Figure 1:**
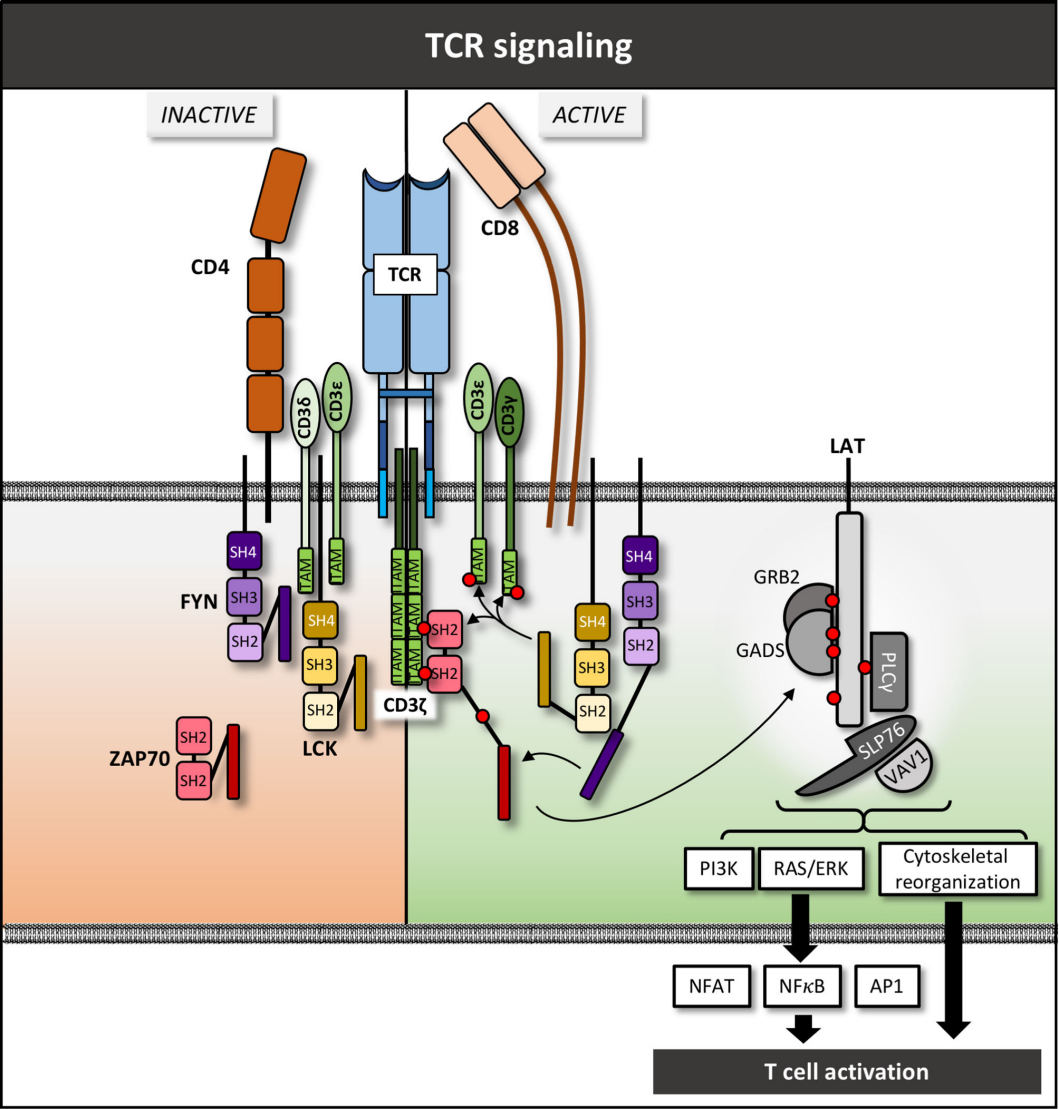
TCR complex and downstream signaling pathway – schematic overview. Red circles denote phosphate groups.

T cell activation, differentiation, and effector functions are tightly controlled by highly specialized and interconnected signaling pathways. Major early mediators of T cell activation include: (1) activation *via* the α and β or *γ* and δ chains of the T cell receptor (TCR) molecules (1); (2) CD3 signal-transduction molecules (2); (3) CD4 and CD8 co-receptors that help stabilize TCR-peptide-MHC interactions (3, 4); (4) early signaling mediators such as LCK, FYN, and ZAP70 (5, 6); and (5) the LAT-signalosome that leads to activation of a myriad of downstream signaling intermediates and pathways (**Figure 1**) (7). These proximal signaling prote13ins interact with myriad of intermediate molecules to ultimately initiate various multiple cellular processes including differentiation and effector function (7). We review the molecules involved in early TCR signaling and the receptors in T cells and consider how mutations or alterations in these molecules contribute to human disease, particularly immunity to cancer. We highlight therapeutic strategies designed to utilize this fundamental knowledge of molecular function for cancer treatment, with an emphasis on novel strategies that are showing early clinical potential. A discussion on T cell based immune therapy would be remiss without mentioning immune checkpoint inhibitors (ICIs) including monoclonal antibodies targeting PD-1, PD-L1 and CTLA4, as well as chimeric antigen receptor (CAR) T cells and tumor-infiltrating lymphocyte (TIL) therapy. These agents have revolutionized cancer therapy, but due to the wealth of literature on these topics, we will not address them directly in this review except as they relate to specific molecules discussed in each section below.

## T Cell Receptor Complex

### TCR: Structure and Function

**Figure 2:**
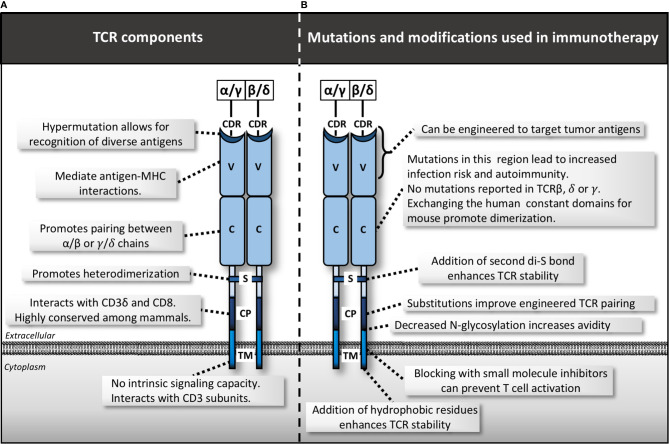
TCR complex structure, mutations and manipulations for immunotherapy. **(A)** Various domains within the T cell receptor (TCR) including the complementarity-determining region (CDR), variable domain (V), constant domain (C), disulfide bond (S), membrane-proximal connecting peptides (CP) and transmembrane (TM). **(B)** Mutations and modifications used in immunotherapy by region.

Unique from all other cell types, T cells express antigen-specific TCRs. The TCR complex is a heterodimer composed of either α/β or *γ*/δ chains, defining two major ”flavors” of T cells. In humans, 95% of T cells are α/β heterodimers while only 5% are *γ*/δ homodimers (**Figure 2**). Within these heterodimers, each chain is comprised of extracellular variable and constant domains, a short linker peptide, and a transmembrane domain (8). The variable domains undergo somatic rearrangement during T cell development and contact the antigenic peptide presented by major-histocompatibility complex (pMHC). The interaction forms the physical basis by which T cells can recognize a myriad of targets (9, 10). The linker and transmembrane domains allow association with additional molecules such as the CD3 chains as well as CD4 and CD8 in their respective T cell types.

### TCR Variable Domain Mutations: Connection to Human Disease and Immunotherapy

As briefly stated above, the variable domain of the TCR genes undergoes somatic recombination early in T cell development, thereby forming the basis for TCR diversity (11). This highly controlled process creates an array of T cells each expressing a unique TCR that can bind varied pMHC complexes. It is estimated that the human T cell repertoire can target on the order of 10 (12) unique specifities (13). Upon recognition of a unique pMHC expressed on target cells, various extracellular and intracellular accessory molecules are recruited to mediate a massive transcriptional shift towards T cell effector functions that allow effective killing of infected, malignant, or altered-self targets (14). Genetic variations in these TCR genes between individuals affect the clonality and diversity of that individual’s T cell repertoire and can lead to disease. Numerous studies have linked intratumoral and peripheral blood TCR clonality and diversity with cancer prognosis and response to various treatment modalities. In cervical cancer fewer TCR clonotypes in sentinel lymph nodes correlated with worse outcomes (15), and in colorectal cancer, patients with metastatic disease harbored less TCR diversity in tumor draining lymph nodes (12). Comparing healthy individuals to a variety of cancer patients, Simnica **et al. found that as people age, their TCR diversity diminishes and that cancer patients have reduced TCR diversity relative to healthy age-matched controls (16). Additional studies in melanoma and pancreatic cancer have shown that increased TCR diversity correlates with improved outcomes after immune checkpoint inhibition (ICI) (17–19), suggesting this treatment modality is reliant on an individual’s T cell repertoire and ability to recognize tumor antigens for its beneficial effect. These data highlight the ability of T cells to recognize and fight cancer and suggest that therapeutic efficacy relies on a diverse TCR pool.

While most anti-tumor studies have manipulated α/β T cells, *γ*/δ T cells have unique features that could be exploited for cancer treatment. In pre-clinical studies, mice lacking *γ*/δ T cells have increased incidence of various cancer types (20–23). This anti-tumor effect is mediated *via γ*/δ T cell recognition of stress-associated molecules often upregulated in cancer such as heat-shock proteins, non-classical MHC molecules, and various phospho-antigens, a process encapsulated under the term the “lymphoid stress-surveillance response.” (24) Furthermore, unique from α/β T cells, *γ*/δ T cells have potent activation responses prior to expansion, and express various NK cell receptors such as NKG2D that further enhance their ability to recognize altered or damaged self-cells (24). The proportion of *γ*/δ T cells infiltrating tumors is predictive of favorable prognosis (25). Among the various tumor types studied, melanoma tumors harbored the highest proportion of *γ*/δ T cells and the presence of these cells correlated with a lower risk of metastasis (26). Interestingly, *γ*/δ T cells are often over-represented in the heterogeneous adoptively transferred cell populations of successful tumor infiltrating lymphocyte (TIL) therapies, again suggesting potent anti-tumor activity (27). However, *in vivo γ*/δ T cell stimulating approaches using IL-2 and bisphosphonates has had underwhelming results (28, 29). Further studies are needed to define optimal *ex vivo* expansion strategies of *γ*/δ T cells, and to piece apart the differential benefit of *γ*/δ and α/β T cell subsets in adoptive transfer approaches.

### TCR Transmembrane and Constant Region Mutations: Connection to Human Disease and Immunotherapy

Aside from the extracellular variable region of the TCR, subtle changes in the extracellular constant and transmembrane (TM) domains can affect the ability of the entire TCR complex to assemble and function (**Figure 2**). The only in-human mutation linked to the TCR α constant (TRAC) domain, is a G to A substitution at the C-terminus of exon 3. This mutation results in a complete lack of α/β T cells, implicating a key role in TRAC in regulating development of this cell type (30). No identified human diseases so far have been linked to mutations in the TM domain, or the TCR β, *γ*, or δ constant domains (TRBC, TRGC, or TRDC), suggesting they are all highly evolutionarily conserved. Indeed, numerous mutational and structural studies have confirmed the essential functions of both the TM and constant domains in assembly of the TCR complex and signal transduction largely via CD3 subunit recruitment and activation (31). More recent studies have only just begun to piece apart the nuanced mechanisms of these interactions (32–34).

New strategies are emerging to manipulate the TM and constant domains of the TCR for therapeutic benefit. For example, the TCR constant domain harbors a disulfide bond that promotes heterodimerization. Adding a second disulfide bond within this region has been shown to enhance TCR stability, signaling and T cell mediated tumor killing in cancer models (35). Adding additional hydrophobic residues to the TM domain enhanced surface expression and T cell avidity, leading to increased anti-tumor T cell activity *in vitro* (36). In mouse and human models, removal of conserved N-glycosylation sites in the TCR variable and constant domains improves T cell avidity and tumor cell recognition (37). Other studies have improved engineered-TCR technology *via* manipulation of constant domains. Exchanging the human constant-region for the murine equivalent (38), or just a 9 amino acid fragment thereof (39), prevents native-non-native heterodimerization. This results in improved pairing of TCR subunits with the desired specificity, enhanced CD3/TCR stability, and increased anti-tumor activity. Introduction of additional cysteine residues in the constant region achieved similar results (40). Finally, new approaches to target transmembrane domains with novel peptides, such as core peptide (CP) targeting of the TM domain of the TCR molecules, has shown early promise in various diseases such as autoimmune disease (41). Similar strategies could be extrapolated to anti-cancer applications. These insights highlight that modulation of anti-tumor immunity should be approached cautiously to maximize the effect on the malignant cells, while preventing detrimental side effects on the host.

### CD3 Subunits: Structure and Function

**Figure 3:**
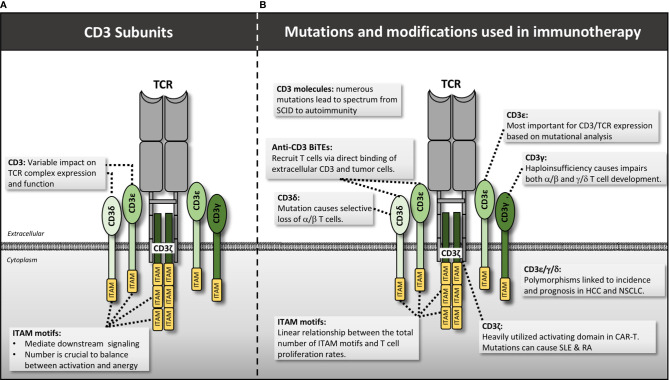
CD3 structure, mutations and manipulations for immunotherapy. **(A)** CD3 molecules interact with the TCR in CD3δ/ϵ and CD3ϵ/γ heterodimers while CD3ζ forms a single homodimer. Each CD3 molecule contains one or more ITAM motif that is essential for downstream signaling. **(B)** Various mutations and genetic engineering approaches alter or manipulate CD3 function thereby impacting T cell activity. HCC, hepatocellular carcinoma; NSCLC, non-small cell lung cancer.

Signaling through the TCR requires interaction with several CD3 subunits, as the TCR chains themselves do not contain intracellular signaling domains (42). There are four CD3 types: CD3ε and CD3δ form a heterodimer that binds to TCRα, CD3ε and CD3*γ* form another heterodimer that binds to the TCRβ chain, and two CD3ζ chains form a homodimer that associates with both TCR α and β chains (**Figure 3**) (31). In the case of the *γ*/δ TCR, two CD3ε/*γ* homodimers are involved in lieu of CD3δ (43). Despite extensive study, the exact geometry and binding sites between these CD3 molecules and the TCR have not been fully elucidated, but likely involve a combination of residues in the constant regions as well as ionizable and hydrophobic residues in the transmembrane regions (44). Signaling itself is mediated through phosphorylation of immunoreceptor tyrosine-based activation motifs (ITAMs) within the CD3 and ζ chains (45).

### CD3 Mutations and Connection to Human Disease

Mutations in the CD3 molecules are associated with an array of human diseases ranging from severe-combined immunodeficiency (SCID) (46, 47) to autoimmune disorders (48). Regarding immunodeficiency, frameshift, nonsense, and splice variant mutations disrupt the ability of the CD3 molecules to be expressed or, if successfully translated, to bind to the TCR molecules to form a functional TCR complex (49). Without functional TCR signaling, T cells fail selection in the thymus, resulting in a complete lack of T cells and severe disease (46, 47, 50–52). More mild immunodeficiencies result in patients where TCR/CD3 is expressed but at much lower levels than normal, impairing T cell activation and function (53–55). The CD3 molecule affected determines the type of deficiency. For example, the CD3δ mutations C202T (that results in a premature stop codon at residue 68), and G to A substitution at position +5 of intron 2, were both found to cause a selective lack of α/β T cells but preserve the *γ*/δ T cell pool because the CD3δ molecule is not included within the *γ*/δ TCR complex (56–58). In contrast, CD3*γ* haploinsufficiency had a larger effect on the *γ*/δ T cell pool, suggesting this molecule is more important in *γ*/δ T cell development in humans (59).

At the opposite end of the spectrum are CD3 mutations that pre-dispose patients to autoimmunity. Of all the CD3 subtypes, mutations in CD3ζ and CD3*γ* harbor the strongest link to autoimmune and inflammatory conditions. Numerous SNPs and splice variants, affecting primarily the 3’UTR, intron 1, and exon 7 of CD3ζ have been associated with pathogenesis in systemic lupus erythematosus (SLE) (48, 60–64) and rheumatoid arthritis (RA) (65). A single SNP in intron 1 (rs858554) has a strong association with both diseases, as well as immune thrombocytopenia (ITP), suggesting a common underlying mechanism (48, 66) possibly via down-regulation of CD3ζ (67). Aside from germline mutations themselves, hypermethylation of the CD3ζ gene has been associated with severe SLE phenotypes and correlates with reduced CD3ζ expression (60). One might predict down-regulation of CD3ζ would decrease T cell activation. However, decreased CD3ζ expression in SLE patients’ T cells was found to result in aberrant recruitment of FcR*γ* to the TCR complex in lieu of CD3ζ (68). Aside from mutations in CD3ζ, CD3*γ* mutations can also result in a range of phenotypes including mild immunodeficiency to autoimmunity. For example, Recio et al. found a common A to T mutation in nucleotide 205 of exon 3 in multiple patients from two different families in Turkey (69). This mutation results in a premature stop codon at residue 69 of the CD3*γ* protein. Despite sharing the exact same mutation, the patients from the first family had severe SCID and died in infancy, while the second patient was largely asymptomatic despite having similarly decreased levels of TCR/CD3 expression. A second study identified a mutation in another Turkish family at position -1 of exon 3 resulting in a premature stop codon at the protein level. This mutation disrupted CD3*γ* expression, resulting in a variety of autoimmune diseases (70). Thus CD3*γ* deficiency results in disparate phenotypes that may be augmented by the larger genetic and environmental context of each individual. To elucidate this further, Rowe et al. studied the function and clonality of T cells from CD3*γ* deficient patients predisposed to autoimmunity. They found that these individuals harbor decreased Treg function, and enrichment of hydrophobic residues at positions 6 and 7 of the CDR3 chain within the variable domain of the TCR, a feature previously linked to auto-reactivity (71). Another detailed analysis of T cell subsets in CD3*γ*-deficient patients revealed impairment specifically in CD8 T cell development, but not CD4 development (72). This shift in the balance of T cell subsets could be responsible for the concurrent immunodeficiency and autoimmunity observed. In general, the heterogeneity of phenotypes resulting from CD3 mutations, particularly CD3*γ*, suggests complex underlying biology that is not fully encapsulated within our current mechanistic models. It will be interesting to apply this foundational knowledge of CD3-chain function to the improved design of engineered TCR’s and CAR’s, and their application in cancer treatment.

The number of ITAM motifs in the signaling domain appears to be key to regulating signal strength and thereby T cell activity. For example, Holst et al. conducted preclinical mouse studies using T cells with different numbers of ITAMs (73). They reconstituted *Rag^-/-^* mice with T cells in which almost every possible permutation of ITAM expression on all CD3 chains (a total of 25 recombinant conditions) was tested for its effect on T cell development and function. Their main conclusions include a quantitative linear relationship between the cumulative number of ITAM motifs in the CD3 complex and T cell proliferation rates. Despite decreased proliferation rates with fewer ITAM’s, they observed that fewer than 7 total ITAMs results in severe autoimmune disease (73), thought due to failed negative selection in the thymus of highly self-reactive T cell clones. An important qualitative difference was observed in mice harboring 6 total ITAMs: autoimmune disease occurred in those with mutated CDζa, the proximal ITAM motif, and CDζc, the distal ITAM motif, but not with mutated intermediate CDζb. No autoimmune disease was observed in mice with wildtype CDζ that lacked ITAMs from CD3δ, *γ*, and ε. It is not known if these phenotypes recapitulate human biology but does imply that individual ITAM’s perform specific roles and that the total number has important implications for T cell function. These types of detailed studies of CD3-subtype roles within the TCR complex will lead to better predictors of disease outcomes and means to genetically target and intervene for clinical benefit.

Notably, a few germline alterations in the CD3 subunits have been linked to cancer. An insertion/deletion polymorphism in the CD3*γ* promoter was linked with increased hepatocellular carcinoma incidence (74), and another (rs3181259T>C) in CD3δ was linked to recurrence in non-small cell lung cancer (NSCLC) (75). SNP rs967591G>A in CD3ε correlated with lower CD3ε expression and shorter survival in NSCLC suggesting a functional consequence could be impaired TCR signaling (76). It is surprising that such little data exists linking CD3 dysfunction with cancer given its essential role in T cell function and thereby adaptive immunosurveillance. Other disease factors in the heterogeneity of human cancers may cloud identification of a CD3 mutation signature in studies based on genetic analysis alone.

### CD3: Connections to Immunotherapy

A variety of therapies have been designed to exploit CD3 subtype functions for cancer treatment. Non-specific stimulation of T cell pools with anti-CD3 antibodies has been used to enhance anti-tumor T cell responses. In TIL therapy, *ex vivo* culture with activating anti-CD3 antibodies along with IL-2 is the preferred method for activation and expansion (77). Compared to α/β T cell activation, CD3 conformational changes do not play as prominent a role in conventional *γ*/δ T cell activation. Nevertheless, binding of anti-CD3 antibodies (78) or Fab fragments (79) to *γ*/δ T cells enhanced tumor killing *in vitro*, suggesting exploiting the CD3 signaling pathway could augment novel anti-tumor properties of this unique cell type.

In CAR-T engineering, derivatives of CD3ζ chains are the favored intracellular signaling moieties incorporated into most CARs (80). Next generation CARs also include intracellular signaling components of costimulatory molecules such as 41BB or CD28 fused to the CD3ζ ITAM domains to further enhance CAR T activation (81). Engineered CAR proteins have been shown to interact with and signal *via* endogenously expressed TCR components (82). Therefore, fine-tuning the intracellular and transmembrane components may result in varied and potentially desirable enhancements of CAR T function. For instance the number and type of ITAM’s impacts the risk of autoimmune disease development in mice (73). With this knowledge in mind, Feucht et al. selectively mutated 1 or 2 ITAM’s of the CD3ζ within a CD19-CD28-CD3ζ CAR and tested the resultant impact on T cell function (83). CAR’s with mutations (X) in the second and third ITAMs (denoted 1XX), were the most efficacious and induced long-term remission in a pre-B acute lymphoblastic leukemia mouse model. Based on the identification of aberrant FcR*γ* recruitment in autoimmune disease when CD3ζ was mutationally defective (84), one might predict that incorporation of the intracellular portion of FcR*γ* into CARs in lieu of CD3ζ domains would result in increased CAR T activation. However, FcR*γ* ITAM domains showed no benefit over CD3ζ when utilized in CAR technology (85, 86), suggesting this synthetic biology does not fully mimic mutation-driven phenotypes observed in nature. Other strategies to enhance CAR T signaling could be inspired by the hyper-activated T cell states observed in autoimmune diseases caused by mutations in other CD3 subunits, and by experimenting with ITAM number and CD3 subunit of origin. However, consideration into the potential risk of chronic activation would have to be investigated.

Another approach that exploits CD3 activity in T cell signaling is to use bi-specific T cell engagers (BITEs). These constructs are comprised of one antibody moiety binding to an antigen of choice and an opposing antibody moiety binding CD3 subunits on T cells (87). The BiTE could be thought of as a soluble CAR, bridging a T cell and target tumor cell. However, in contrast to CARs, BiTEs are still reliant on endogenous CD3 expression in the T cells they recruit. BiTEs have been shown to induce responses in polyclonal populations of CD4 and CD8 T cells (88), and do not have to be custom made for each patient. Currently most bind to the extracellular component of CD3ε (88). Based on our above appreciation for the heterogenous involvement of various CD3 subunits in T cell function and disease, targeting other CD3 subunits could be used to fine-tune desired T cell recruitment with BiTEs. BiTE efficacy is in part limited by specific tumor cell phenotypes, such as the expression of sialophorin which limits T cell to tumor cell adhesion (89), and by similar side effects of CRS and neurotoxicity observed in CAR Therapy (90, 91). One study also identified a polymorphism in the CD3ζ chain (SNP rs2949655) that correlated with reduced cytotoxicity in response to BiTE treatment (92), demonstrating how CD3 mutational profiling could be used to help guide personalized treatment approaches targeting this pathway. Further improving our understanding of CD3 subunit function and signaling will help elucidate additional strategies for therapeutic intervention.

### CD4 and CD8: Structure and Function

The co-receptors CD4 and CD8 are essential to T cell development and formation of a functional TCR-MHC synapse (93). They are considered co-receptors because they stabilize MHC-antigen-TCR complexes and contribute to the functions of CD4+ helper T cells and CD8+ cytotoxic T cells without direct antigen binding (94, 95). CD4 is comprised of four extracellular Ig domains that bind to MHC-II. CD8 is a dimer comprised of either a CD8α homodimer or a CD8α and CD8β heterodimer (96, 97). These extracellular domains are attached to a long extracellular stalk domain that, by means of differential sialylation/glycosylation, helps regulate CD8-MHC-I binding affinity (98). CD8αα is found on *γ*δT cells, intestinal and dermal intra-epithelial T cells, as well as NK cells (99). CD8αβ, on the other hand, is primarily expressed on conventional cytotoxic CD8 T cells (100). Crystal structures have shown CD4 binds at membrane-proximal α2 and β2 domains of the MHC-II molecules at residues conserved between the different MHC-II types (101), while CD8 binds mainly at the α3 domain of MHC-I (102). MHC-II/CD4 or MHC-I/CD8 binding occurs at much lower affinity than MHC/TCR, presumably to help calibrate appropriate T cell selection during thymic development and avoid autoimmunity (100). Both CD4 and CD8 have transmembrane domains that contribute to the formation of their respective TCR complexes, as well as intracellular domains that associate with LCK to facilitate intracellular signaling.

### CD4 and CD8 Mutations: Connections to Human Disease and Immunotherapy

The only known polymorphisms in CD8α (p.Gly111Ser) associated with human disease affect functional expression of the CD8α molecule, resulting in compete lack of CD8 T cells (103). No other mutations or even polymorphisms in the CD4 molecule or CD8β have thus far been linked to human disease, likely because each has such refined and essential functional requirements for T cell function. In mice, studies have shown that missense or non-functional mutations of CD4 and CD8 result in lack of either CD4 or CD8 T cell subtypes due to failure of thymic selection (104, 105).

The use of CD4 and CD8 as markers for specific T cell subsets and their functions has profound implications to tumor immunology and immunotherapy. Within the tumor microenvironment, increased T cell infiltration, specifically of CD8 cytotoxic T cells, and high CD8/FoxP3 ratios correlate with better overall survival in multiple cancer types (106). Th1 phenotypes of TILs correlate with improved outcomes (107). In adoptive cell therapy, it was thought that the CD8 component of TILs was the most important due to the known cytotoxic capability of CD8 T cells, with CD4 T cells playing a merely supportive role (108, 109). Indeed, in melanoma patients a higher frequency of CD8 T cells amongst the infused cells correlated with better responses (110). However, CD4 T cells have been shown to exert anti-tumor effects through largely unknown and likely multivalent mechanisms (111). Additionally, recent evidence has shown CD4 T cells can acquire cytotoxic capabilities in the presence of IL-2 and mediate direct tumor cell killing (112). For CAR T cell therapy, a combination of CD4 and CD8 T cells in a defined 1:1 ratio appears to be most efficacious (113). Current TIL therapy regimens use *ex vivo* IL-2 expansion, but do not select for T cell subsets prior to re-infusion (77). More detailed studies of CD4 and CD8 TIL subsets and means to expand and enhance their function *ex vivo*, as well as the optimal balance of CD4 and CD8 cell types in adoptive cell therapies, are required.

Various approaches to target CD4 have been applied or are in development for immunotherapy. CARs targeting CD4 have shown promise in pre-clinical models of peripheral T cell lymphomas (PTCLs) (114). Anti-CD4 antibodies have been used in patients with PTCLs as well as cutaneous T cell lymphomas with some early clinical benefit (115–118). Anti-CD4 antibodies have also shown benefit in non-hematologic malignancies *via* depletion of anti-inflammatory CD4 T cell subsets including Tregs, thereby allowing for enhanced proliferation of anti-tumor CD8 cytotoxic T cells (119). This approach is further bolstered with the addition of immune-checkpoint blockade (120) and is now being applied in early clinical trials (121).

Aside from using CD4 and CD8 as cell-specific markers, genetic manipulation of these molecules offers therapeutic potential. In our lab, a synthetic construct that fused the CD8α extracellular domain to a MyD88 intracellular domain, normally downstream of innate immune receptors, resulted in enhanced anti-tumor CD8 T cell function in mouse models (122). Another group demonstrated increased MHC-I binding affinity when sialylation of core 1 O-glycans on the CD8 stalk region was reduced either through neuraminidase treatment or mutation of ST3Gal-I sialyltransferase mutation (123). Through a phage display approach, Wang et al. found the substitutions Gln40Tyr and Thr45Trp in CD4 resulted in almost 500 fold increase in MHC-II binding affinity (101). Future studies are need to determine if other alterations in CD8 or CD4 extracellular domains could be used to further stabilize the TCR’s interaction with low affinity tumor antigens and thereby improve anti-tumor CD8 and CD4 effects respectively. Furthermore, docking topology of self-reactive TCR-MHC-II complexes in autoimmune T cell types is different than that in non-self-reactive T cells, and is likely influenced by CD4 molecule binding (13). One could envision that modulating CD4-TCR-MHC topologies could in a similar manner enhance T cell responses to rare self-like tumor-associated antigens (TAA). In conclusion, the CD4 and CD8 molecules are well-established markers for T cell subsets, their potential as therapeutic targets show early promise, and alteration in their function and/or binding activity warrants continued exploration.

## Early Downstream Signaling Intermediates: LCK, FYN, ZAP70

### LCK: Structure and Function

**Figure 4:**
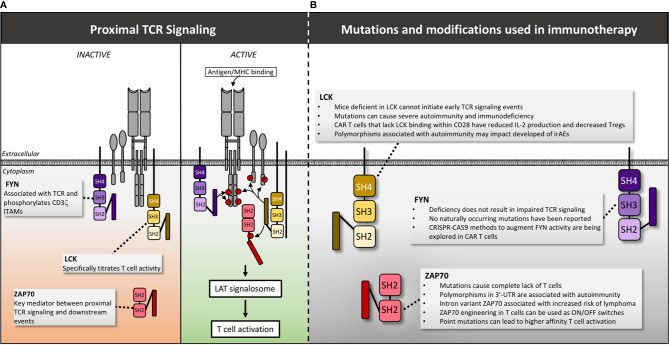
LCK, FYN and Zap70 structure, mutations and manipulations for immunotherapy. **(A)** Following antigen/MHC binding, LCK is recruited first to the TCR, initiating phosphorylation of ITAMs within the CD3 intracellular domains. Subsequently FYN is recruited and assists in phosphorylation of CD3ζ ITAMs. LCK then phosphorylates the linker sequence of ZAP70 causing a conformational change that actives the kinase activity of ZAP70 permitting additional downstream signaling. **(B)** The impact of mutations and genetic manipulation of LCK, FYN and ZAP70 are detailed. Red circles denote phosphate groups.

Besides the molecules within the TCR signaling complex, many additional proximal signaling molecules also contribute to overall T cell function and hold potential as therapeutic targets. The precise events that occur following TCR activation remain controversial, but current data suggests that early proximal signaling events are largely mediated by the Src family kinases LCK and FYN (124). Upon antigenic stimulation of a T cell, pre-activated LCK is recruited first to the TCR, initiating phosphorylation of ITAMs within the CD3 intracellular domains (**Figure 4**). Subsequently, additional LCK bound to the CD8 and CD4 co-receptors localizes to the immune synapse, enhancing TCR-pMHC binding and enabling ZAP70 recruitment and activation (125–128). Additionally, LCK deficient mice have severely impaired T cell development (129–132) and LCK contributes to a T cells’ ability to titrate its activation level based upon the affinity of the TCR-pMHC interaction. This graded signaling response is largely regulated via distinct patterns of ITAM phosphorylation executed predominantly by LCK (133). LCK activity also modulates T cell differentiation (134, 135), CD28 costimulatory signaling (136), and even cell death (137). Given its critical role in a variety of T cell functions, it is not surprising that LCK activity is tightly regulated to maintain immune homeostasis. Consequently, genetic variations and mutations that alter the function of LCK have profound implications for the development of cancer and immune-based therapies.

### LCK Mutations and Connections to Human Disease

A homozygous missense mutation in a hydrophobic region of the catalytic domain (c.T1022C) has been identified in children presenting with severe recurrent infections, autoimmune manifestations and panniculitis. This mutation is associated with reduced CD4 and CD8 expression, impaired TCR activation, decreased Treg levels and expansion of an oligoclonal *γ*/δ T cell population (138). Additional splice mutation variants of LCK have been reported and correlate with impaired LCK function and immune dysregulation. Patients with these variants present with a range of clinical syndromes including epidermodysplasia verruciformis and recurrent bacterial infections (mutation: c.188-2A>G) (139), common variable immunodeficiency (CVID, mutation: lack of exon 7) (140), and severe combined immunodeficiency (SCID, mutation: lack of exon 7) (141). Mutations that delete a C-terminal regulatory tyrosine within LCK increase LCK activity resulting in sustained T cell activation and oncogenesis in mice (142), as well as increased IL-2 production independent of antigenic signaling in humans (143). In addition, a chromosomal translocation t(1;7)(p34;q34) has been identified in patients with T cell acute lymphoblastic leukemia (ALL) (144). This translocation also increases LCK activity and the degree of elevated activity is correlated with breakpoint location and disease severity (144). These findings are intriguing from a T cell engineering perspective given that modulating LCK function, even *via* changes in single residues, has significant impact on T cell function. However, based upon the stark clinical phenotypes observed among patients, it is clear that extreme caution is warranted when considering genetic manipulation of this crucial protein.

### LCK: Connections to Immunotherapy

Understanding LCK activity and genetically altering this gene still holds promise for several therapeutic approaches in oncology. In particular, LCK modulation is an important area of study for CAR T cell treatment of solid tumors. For example, CAR T cells have been engineered with a deleted LCK-binding motif within CD28 (ΔCD28). In mice, these second generation ΔCD28/CD3ζ (145) and third generation ΔCD28-4-1BBζ CAR T cells (146) show reduced IL-2 production and improved tumor control in the presence of Tregs. Clinically, a patient with malignant pleural mesothelioma was treated with anti-FAP ΔCD28/CD3ζ CAR T cells and experienced stable disease for 1 year, suggesting that ΔCD28/CD3ζ CAR T cells may have contributed in controlling his disease (147). However, this finding is currently anecdotal and other studies suggest that the effect of ΔCD28/CD3ζ on CAR T cell function may depend upon the immunosuppressive mechanism within the tumor microenvironment (TME) (148). For instance, tumor models with high TGFβ within tumor tissue require an intact LCK motif within the CD28/CD3ζ CAR receptor in order to overcome TGFβ-mediated suppression (148). Therefore, patient stratification by TME immune profiles could identify patients who would benefit from ΔCD28/CD3ζ CAR T cells. Synthetic LCK modulation in CAR T cells has other therapeutic implications as well given that LCK deficient cells are resistant to activation induced cell death (149) and LCK is involved in PD-1 induced inhibition (150).

It is also important to consider the impact of LCK mutations in the context of immune checkpoint inhibition (ICI). For example, the LCK SNP rs10914542 G allele impairs TCR activation (151), suggesting that patients harboring this allele may be less likely to mount a robust response following ICI therapy. Conversely, a single amino acid variant in LCK (p.G85W of exon 4) was associated with autoimmune diseases including Sjogren syndrome, SLE and RA, suggesting that patients with this variant could be at increased risk of developing autoimmune complications in the setting of ICI (152). All together, these data suggest that artificial regulation of LCK or screening for LCK variants could inform immune-based therapeutic strategies for cancer.

### FYN: Structure and Function

FYN is another Src family tyrosine kinase involved in proximal TCR signaling, however, the precise roles of this protein are less understood than LCK. FYN also associates with the TCR and is involved in phosphorylation of CD3ζ ITAMs. However, LCK deficiency causes a much more dramatic phenotype than FYN deficiency, suggesting that FYN is not required for T cell activation (126). FYN interacts with many additional binding partners including PI3K (153, 154), lymphocyte-specific scaffold protein adhesion and degranulation-promoting adaptor protein (ADAP) (155), phosphoprotein associated with glycolipid-enriched membranes (PAG) (156), signaling lymphocyte activation molecule (SLAM) and others (157). These interactions collectively enable a diverse breadth of functions ranging from T cell activation to anergy (158). It is therefore reasonable to hypothesize that alterations in FYN function could modulate T cell activity in a variety of ways that could be exploited for therapeutic purposes.

### FYN: Mutations and Connections to Immunotherapy

To date, no naturally occurring mutations in FYN have been linked to disease, suggesting that either existing genetic variants are relatively benign, or they cause lethality. Despite this ambiguity, modulating FYN activity remains an active area of interest in T cell engineering. For example, in a recent review by Thakar et al., the authors propose that inhibition of the FYN-ADAP pathway using CRISPR-CAS9 could provide a unique means of selectively downregulating cytokine production by CAR T cells without impairing cytotoxicity (159). This approach could be used to reduce the severity of cytokine release syndrome, a dangerous complication of CAR T cell therapy (159). Inhibiting FYN activity may also enhance T cell migration. A study by Schaeuble et al. reported that inhibition of FYN with the small molecule SU6656 promoted enhanced CCR7-driven migratory function of nonactivated T cells *in vitro* (160). CCR7 is expressed by both central memory T cells (T_CM_) and T memory stem cells (T_SCM_), both of which are promising substrates for both CAR T cell (161, 162), and transgenic T cell therapy (163). Therefore, inhibiting FYN activity in genetically engineered T cells could enhance their migratory capacity prior to activation within either a tumor-draining lymph node or the TME.

Alternatively, activating some functions of FYN could improve cellular based immunotherapy. One study found that deletion of FYN in a mouse model promotes differentiation of CD4+ T cells towards a Treg phenotype and away from a Th17 phenotype (164). While speculative, this suggests that activating Fyn could promote a Th17 phenotype. Some data suggest that Th17 CD4+ T cells have superior anti-tumor function and improved persistence as compared to Th1 cells in adoptive cell therapy settings (165–167). Therefore, FYN modulation in CD4+ T cell engineering approaches may not only reduce Treg induction but could also promote a Th17 phenotype. Increasing FYN activity has other potential beneficial implications. The SH2 domain of FYN binds Tim-3, promoting T cell activation and increasing cytokine production (168), a surprising finding as Tim-3 is classically associated with T cell exhaustion. Increased FYN-Tim-3 binding *via* genetic modification could shift the balance of Tim-3 activity towards T cell activation and away from exhaustion (168). In contrast to the T cell activating function of FYN, FYN association with PAG and c-cbl can disrupt canonical TCR signaling and promote T cell anergy under certain conditions (156, 169–173). These findings indicate that FYN has highly varied and even paradoxical effects on T cell activity depending upon its binding partners. Importantly, the binding site for these various partners are not all known. Therefore, further characterization of the specific binding locations could enable genetic alterations to precisely tailor FYN activity and improve active immunotherapeutic approaches. Regarding passive immunotherapeutic strategies such as ICI, FYN-activating signatures have been associated with lupus nephritis, an autoimmune condition (174), loosely suggesting that increased FYN activity might increase the risk of autoimmune complications following ICI. However, no polymorphisms or genetic variants have been associated with autoimmunity thus it is unlikely that mutational profiling of FYN would predict development of immune related Adverse Events (irAE). Overall, further characterization of FYN function, binding sites and binding partners is warranted and may provide opportunities for various T cell engineering strategies in the future.

### ZAP70: Structure and Function

Once LCK and FYN phosphorylate ITAMs on the CD3 molecules, the next step in TCR signal transduction involves binding of ζ-chain-associated protein kinase of 70 kDa (ZAP70) (45). Distinct from the Src family kinases described above, ZAP70 along with SYK are the two prominent members of the Syk family of kinases (175). ZAP70 is comprised of an auto-inhibited kinase domain and two amino-terminal SH2 domains that bind doubly-phosphorylated ITAMs (176) Upon ITAM binding, ZAP70 undergoes a conformational change that results in additional phosphorylation of residues in the second linker sequence by LCK. This relieves inhibition of the kinase domain and results in downstream signal propagation (177).

### ZAP70 Mutations and Connections to Human Disease

Various ZAP70 mutations have been shown to cause a severe form of immunodeficiency characterized by complete lack of functional T cells (178). Most of these mutations affect the kinase domain (179–182), or lead to loss or destabilization of the protein transcript altogether (183–185). Syc can take the place of ZAP70 in T cell signaling when the latter is impaired, allowing for some CD4 cells to survive thymic selection, but these Syc+ZAP70- T cells are defective in IL-2 production and proliferation and provide aberrant help to B cells for antibody class switching (182, 185). In contrast, polymorphisms in the ZAP70 coding region or 3’-UTR have conversely been associated with autoimmune disorders including psoriasis and type 1 diabetes (rs17695937) (186, 187), inflammatory bowel disease (IBD, rs13420683) (188), and RA (rs2278699) (189). Finally, an intron variant of ZAP70 (rs7425883) is associated with a decreased risk of developing non-Hodgkin lymphoma (190), and aberrant elevated expression of ZAP70 in B cell CLL cells correlates with enhanced BCR signaling in the leukemic cells and poorer prognosis (191, 192). Mouse studies recapitulating some of these autoimmune or immunodeficient phenotypes have demonstrated point-mutations in the second SH2 domain, interdomain B, or paired mutations in the kinase region lead to aberrant thymic selection more permissive of higher affinity self-reactive clones, and quantitative differences in TCR signaling (193–195). Of particular interest, the tyrosine residues at positions 292, 315, and 492 have been shown to play a negative regulatory role when phosphorylated, and their mutation to phenyalanine (which prevents phosphorylation), allowed for T cell hyperactivation (196). These findings suggest that ZAP70 could be used not only as a prognostic marker of disease but also a therapeutic target.

### ZAP70: Connections to Immunotherapy

Due to its upstream role in TCR signaling, ZAP70 has been gaining interest in the world of cancer-immunotherapy. Of greatest potential, engineered ZAP70 constructs have been designed to function as on/off switches to control T cell responses. In one approach, a larger analog of the kinase inhibitor PP1 was used to selectively inhibit an engineered ZAP70 with altered inhibitor binding affinity, resulting in impaired catalytic activity (197). Interestingly, Treg function was not affected by this approach, suggesting unique non-catalytic activity of ZAP70 is functioning in Tregs (198), and that therapeutic application of this design would not affect tolerance and protective roles of adaptive immune cell types. This analog sensitive ZAP70 could be employed to turn off unwarranted activation of adoptively transferred T cell therapies. Other investigators have developed a tetracycline-inducible ZAP70 gene promoter allowing for selective turning on of ZAP70 transcription (199). This has so far only been used in studies designed to assess the role of ZAP70 in thymic selection, and clinical application may be impaired by the need for tetracycline infusion and difficulty regulating expression levels once turned on. More recently, a dual small-molecule gated ZAP70 has been created *via* fusion of the analog-sensitive ZAP70 to the ligand binding domain of the estrogen receptor (200), allowing for both on and off signals. *In vitro*, this new ZAP70 construct could be controlled on a minute-by-minute timeframe and regulated calcium flux and CD69 expression levels. However, cytokine production was impaired in the “on” configuration, thus more refinement is required before clinical application (200). Future studies could examine the role of specific ZAP70 point mutants, such as those identified above that result in autoimmune phenotypes, in regulating adoptive T cell therapy efficacy. Additionally, the differential role of Syk and ZAP70 with regards to TCR signaling is still an area of inquiry with some conflicting evidence regarding the potency of the two molecules for T cell activation (182, 185, 201, 202). Although early studies of CAR design have favored CD3ζ and ZAP70 dependent designs, recruiting the Syk tyrosine kinase in lieu of ZAP70 may have some benefit in specific contexts. Finally, CXCR3-mediated T cell chemotaxis was shown to be dependent on ZAP70 and was impaired by TCR signaling (203). Modulating ZAP70 crosstalk between these two important pathways could affect the ability of T cells to infiltrate the tumor microenvironment and maintain TCR signals and resultant activation states. Collectively, this early evidence demonstrates artificially controlling ZAP70, or variants of ZAP70 with different signaling thresholds, could be powerful tools for cancer immunotherapy.

## The LAT Signalosome: LAT, Binding Partners and Downstream Pathways

### LAT: Structure and Function

**Figure 5:**
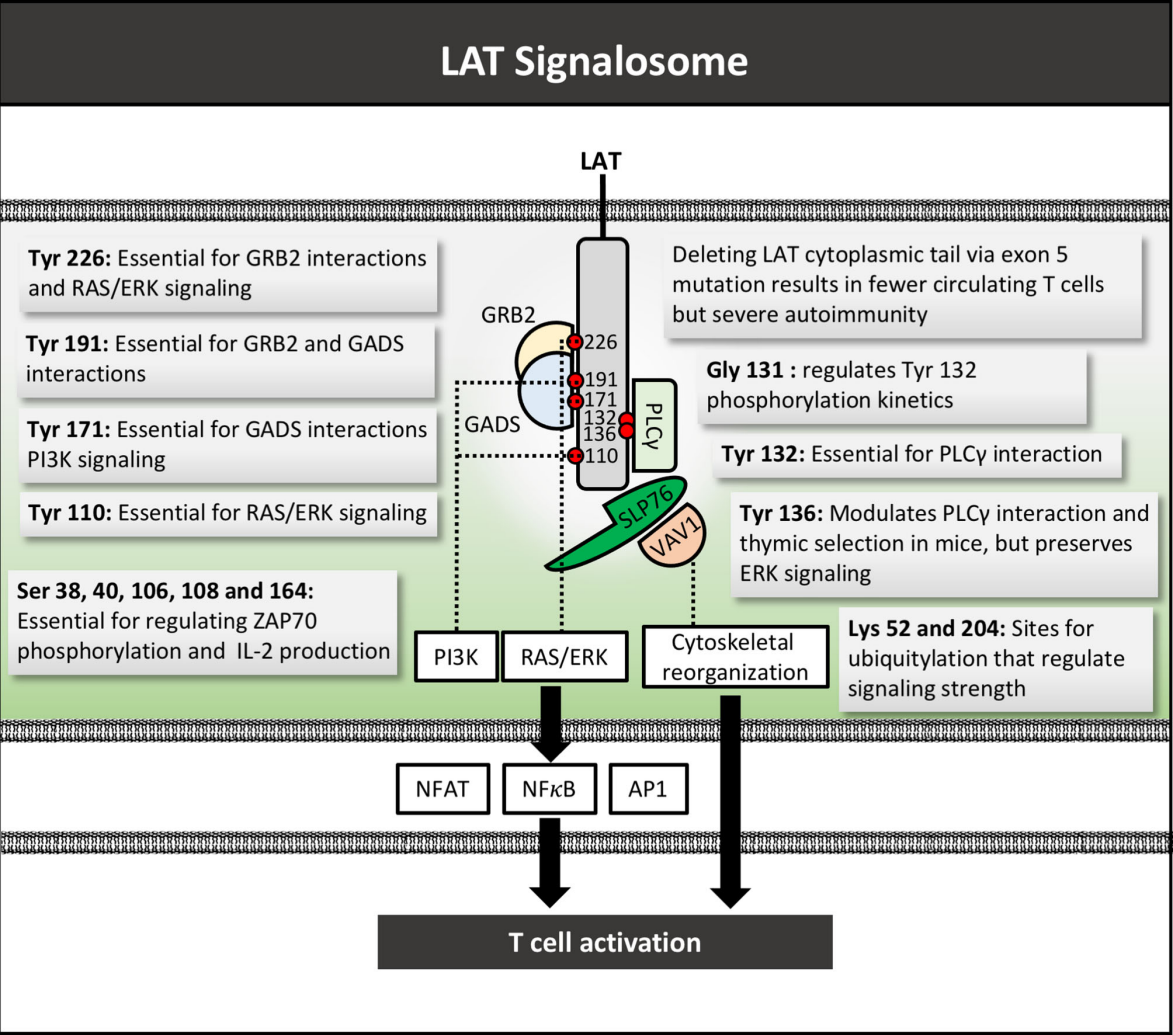
LAT signalosome structure and function. LAT signalosome schematic structure with function of key tyrosine residues depicted by their respective numbers, as well as mutations and respective phenotypes. Several key LAT signaling partners are depicted. Red circles denote phosphate groups. Tyr, Tyrosine; Gly, glycine; Ser, serine; Lys, Lysine.

LAT serves as a major junction point in TCR signaling, forming a nexus between the early antigen-recognition machinery and a multitude of downstream pathways (**Figures 1** and **5**) (204). Phosphorylation of LAT at multiple intracellular tyrosine residues by ZAP70 is a key link between TCR antigen recognition and the transcriptional paradigm shift of T cell activation (205). Due to its assembly of numerous signaling molecules, LAT has been referred to as the central platform for the “LAT signalosome.” (206) LAT is comprised of minimal extracellular and transmembrane domains, and an extensive cytoplasmic region with numerous phosphorylation and protein binding sites (207).

### LAT Mutations and Connections to Human Disease

Mutational mapping has allowed identification of tyrosine-phosphorylation residues required for LAT to associate with individual signaling partners. For example, mutation of tyrosine 132 in human T cells results in defective binding to PLC*γ*-1 (208), tyrosine 171 was essential for PI3K activation (209), tyrosines 110 and 226 are required for ERK activation (208), 171 and 191 required for Gads binding, and 171, 191, and 226 together are required for Grb2 binding (**Figure 5**) (210). Aside from tyrosine residues, study of the human Jurkat T cell line identified 11 serine residues in the cytoplasmic domain that may be key to signal propagation. Cells expressing LAT with S->A mutations at serines 38, 40, 106, 164, and 180 exhibited decreased PLC*γ*-1 and SLP-76 binding, reduced IL-2 production, but increased ZAP70 phosphorylation (211). The impact of each individual serine residue has yet to be elucidated. A recent study identified as many as 90 putative binding partners for LAT, suggesting the myriad of established roles for residues in this molecule may yet underestimate the importance of LAT in T cell activation (212).

Studies in mice have allowed mechanistic elucidation of the key function of LAT and its structure in T cell development and mature T cell functions (213–215). Completely blocking LAT expression or function in mice led to impairment in T cell development. Mutation of the distal 4 tyrosine residues of LAT to phenylalanine mimicked the phenotype of LAT-/- mice, in which T cell development is completely blocked at the double-negative 3 (DN3) stage (215). Selectively knocking-out LAT expression after the DN3 stage led to impaired development of single positive T cells in the thymus and periphery (216). Conversely, mutations or deletions of LAT in mature post-thymic T cells altered but did not completely impair T cell functions, and even led to autoimmunity and aberrant lymphoproliferation, implicating LAT in not only T cell activation but also regulation (217, 218). In one study, a mutation in tyrosine 136 and impaired PLC*γ* binding led to MHC-independent constitutive Th2 T cell activation and T cell dependent B cell hyperproliferation and antibody production, ultimately resulting in systemic autoimmune disease (219). Others showed that the same mutation resulted in disruption of thymic positive and negative selection (220). Similarly, a third study showed that mutation of tyrosine 136 blocked PLC*γ* dependent functions, but allowed for continued ERK signaling, first causing impaired T cell development but later causing a lymphoproliferative disorder (221). The mechanism of negative regulation *via* LAT could be mediated *via* association with Grb2, a known inhibitory signal mediator (222), or through binding and inhibiting the active form of LCK (223). Together these results implicate LAT in both T cell thymic selection as well as subsequent immune regulation, and that complex interactions between LAT and its multiple binding partners maintains a balance between T cell activation and inhibition.

In humans, mutations or polymorphisms in LAT recapitulate the range of mouse phenotypes ranging from autoimmunity to immunodeficiency (224). Higher expression levels of LAT (as assessed by qRT-PCR and flow cytometry) were correlated with severity of aplastic anemia, however it was unclear how this over-expression was regulated or if this was a cause or consequence of disease (225). Loss of the cytoplasmic tail of LAT due to a mutation in exon 5 resulted in immunodeficiency characterized by a decrease in circulating T cells, but simultaneous severe autoimmunity (226). T cells in these patients were still able to induce calcium influx and NF-κB activation, but had aberrant ERK signaling (226). The heterogeneity of disease resulting from LAT-deficiency has led some to distinguish LAT-dependent pathology due to immune hyper-activation from true autoimmunity (227). The mouse studies above do suggest LAT also plays a role in regulating thymic selection and can lead to true autoimmune T cells. A similar role in humans has not been entirely ruled out. These studies underscore the fine balance between pro and anti-inflammatory processes that evolved to maintain homeostasis within the immune system, and that LAT is a key regulator of this balance in T cells.

### LAT: Connections to Immunotherapy

As of now, no immunotherapies have modulated LAT to enhance anti-cancer T cell responses. Based on our above understanding, it is possible that changes in LAT would lead to too many off target effects of T cell-based therapies, or persistent non-specific inflammation that would be detrimental to the host. However, careful alterations could also increase T cell activation in response to weak or rare neo-antigens or help adoptively transferred T cells maintain activation states in solid tumors. These varied effects could be achieved by selectively mutating key tyrosine or serine residues within the cytoplasmic tail. Additional modulations could include replacing lysine with arginine residues, which was shown to decrease ubiquitylation and enhance T cell signaling (228), or mutating residues neighboring key tyrosines to alter phosphorylation kinetics (229). Furthermore, the use of LAT instead of CD3ζ as the intracellular signaling component of CARs could be considered. Extensive pre-clinical testing would be required to ensure any increased T cell activation caused by LAT modulation would not result in intolerable or dangerous side effects and means to kill or turn off such cells should be incorporated as a safety mechanism.

### LAT Binding Partners and Downstream Signaling

LAT serves as a major hub after which TCR signaling networks with many other pathways in T cells, such as those downstream of chemokine and cytokine receptors and costimulatory molecules (204). As a consequence, many highly complex, and tightly regulated interactions take place including activation of canonical MAPK, NF-κB, Ca^++^-mediated signaling pathways that ultimately regulate the nuanced transcriptional profiles characteristic of each T cell phenotype (**Figure 5**) (217, 230). Abnormalities in any one of the many proteins involved in these processes can lead to aberrant T cell phenotypes. Some of the best described interactions between LAT occur with ITK, PLC-*γ*1, Grb2, SLP-76, VAV1, and FYB1 (230). Most of these are activating, but the interaction with Grb2 is generally inhibitory to T cell activation, as briefly mentioned above (222). As an example of the spectrum of diseases caused by these downstream proteins, gain of function of ITK is common in T cell lymphomas (231), loss-of-function mutations are associated with lymphoproliferative diseases (232), full ITK deficiency has been linked to idiopathic CD4+ T cell lymphopenia (233), and SNPs in ITK have been associated with asthma (234). Polymorphisms in FYB as well as low levels of LAT-PLC-*γ*1 interactions have also been implicated in susceptibility to asthma, perhaps due to skewing of T cell polarization towards an aberrant Th2 phenotype (235, 236). VAV1 has strong links to oncogenesis in various tissues (237–239), with mutations causing disruptions in multiple pathways including Ca^++^ signaling (240, 241). It is important to note that many of these downstream mediators are involved in signaling in other immune cell types besides T cells, and receptor driven pathways other than the TCR pathway even within T cells. For example, PLC-*γ*1 is involved in FcεRI-mediated mast cell degranulation, thus its association with asthma maybe more due to effects on mast cells as opposed to T cells (242). FYB is also expressed in many other immune cell types of both the innate and adaptive system with diverse functions, making cell-type or pathway specific conclusions difficult (243). Any targeting of these molecules to alter T cell function must take into account that not only T cells or the TCR pathway may be affected.

Given the complexity of this stage of T cell activation, no therapies targeting these downstream networks have yet been approved in cancer immunotherapy. However, a few groups have begun to explore distal TCR signaling modulation to benefit immunotherapy. For instance, knocking out ADAP in adoptively transferred T cells reduced PD-1 expression and increased anti-tumor efficacy in mice (244). Other pre-clinical studies have shown that Cis-mediated inhibition of CD8 T cells functions by down-regulating PLC-*γ*, and targeting this pathway could increase potency of CARs and other adoptive therapies (245). Outside the context of immunotherapy, directly targeting some of these molecules could also have direct anti-neoplastic effects. Much more cell-type specific, and pathway specific studies are needed before targeting these downstream mediators can be used safely and effectively in treatment of human cancers.

## Conclusions

The TCR proximal signaling pathway that regulate T cell function comprise a complex cascade of interactions involving numerous extracellular and intracellular proteins with unique functions. Perturbations such as polymorphisms and mutations in each contribute to a myriad of human diseases ranging from immunodeficiency to autoimmunity, and some even have significant contributions to various malignancies. Understanding the nuanced role of each molecule has allowed for the design of immunotherapies to take advantage of the significant involvement of TCR signaling and cytokine activation of T cells in cancer. The genetic alterations described throughout this review are summarized in **Table 1**.

**Table 1:** Mutations and molecular alterations in the TCR pathways.

Gene	Mutation/Alteration	Structural Outcome	Immunological Outcome	Clinical Outcome	Therapeutic Relevance	Reference
**TCRα**	G>A at the C-terminus of exon 3 (TRAC domain), chromosomal region 14q11.2	Partial loss of the connecting peptide domain and abolition of the transmembrane and cytoplasmic domains of the TCRα chain - impaired TCR complex assembly	Complete lack of α/β T cells	Patients would be excluded from allo-ACT therapies due to lack of endogenous T cells	Patients lacking T cells could more likely benefit from allo-ACT strategies by demonstrating reduced host v. engrafted T cell responses	(30)
**TCR^**	p.T48C on α chain and p.S57C on β chain	Creation of second disulfide bond within constant domain	Enhance TCR stability and signaling and T cell mediated tumor killing in cancer models Possibility of expedited T cell exhaustion due to increased TCR strength	Enhanced recognition of lowly expressed or weakly immunogenic tumor antigens	Enhance T cell mediated immunotherapy Engineering this mutation into tumor-reactive TCR to improve antitumor responses	(35)
**TCR^**	Substitution of leucine/isoleucine residues for 7 hydrophilic residues in TCRα and 10 hydrophilic residues in TCRβ	Increased hydrophobic interactions in the transmembrane domain	Enhanced TCR surface expression and T cell avidity; increased anti-tumor T cell activity *in vitro* Possibility of expedited T cell exhaustion due to increased TCR strength	Enhanced recognition of lowly expressed or weakly immunogenic tumor antigens	Enhance T cell mediated immunotherapy Engineering this mutation into tumor-reactive TCR to improve antitumor responses	(36)
**TCR^**	Mutating N of the N-glycosylation motif (N-X-S/T) to a glutamine. TCRα: position 84C, 90 and 113 on vα3. TCRβ: position 1.3, 84.5 and 113 on vβ3.	Decreased N-glycosylation of extracellular constant domain	Increased TCR avidity	Enhanced recognition of rare tumor antigens by TCR-pMHC and improved anti-tumor immunity	Enhance T cell mediated immunotherapy Engineering this mutation into tumor-reactive TCR to improve antitumor responses	(37)
**TCR^**	Exchange human constant region with murine equivalent or 9 amino acids from murine constant region	Prevents native-non-native heterodimerization	Improved pairing of TCR subunits, enhanced CD3/TCR stability, increased anti-tumor activity	Improved outcomes in adoptive transfer approaches using engineered TCRs	Improved outcomes in adoptive transfer approaches using engineered TCRs	(38–39)
**TCR^**	p.T48C on α chain and p.S57C on β chain	Improved engineered TCR chain pairing, decreased pairing with native TCR chains	Improved engineered TCR T cell tumor antigen recognition and thereby anti-tumor activity	Improved outcomes in adoptive transfer approaches using engineered TCRs	Improved outcomes in adoptive transfer approaches using engineered TCRs	(40)
**TCR^**	Core peptide targeting of TM domain	Interrupt cohesive interactions between proteins and with TM lipids	Blocks T-cell mediated killing	Prevent autoimmunity	Treatment of autoimmune diseases, could be extrapolated to cancer therapies Use as a strategy to inhibit unwanted activity of TCR-engineered or TIL therapies	(41)
**CD3D and CD3E**	c.279C>A, c.202C>T leading to p.C93X and p.R68X nonsense codons of CD3D respectively. 2-bp deletion at nucleotide 128 of exon 5 of CD3E leading to frameshift and nonsense codon at residue 56.	Truncation of the extracellular domains of CD3D and CD3E respectively	Total lack of CD3+ thymocytes	SCID	Patients lacking T cells could more likely benefit from allo-ACT strategies by demonstrating reduced host v. engrafted T cell responses	(46)
**CD3Z**	38 SNPs in intron 1		Disrupt CD3 expression or ability of CD3 to bind TCR	Association with SLE	Potential use as clinical marker to predict efficacy or development of autoimmunity of ACT T cell therapies	(48)
**CD3E**	Deletion of 173T in exon 6	Premature stop codon	Lack of T cells	SCID		(50)
**CD3G**	A>G mutation in initiator codon and G>C mutation at intron 2-exon 3 splice site	Severe truncation or lack of CD3G translation	Low level expression of TCR on T cells	Spectrum from SCID to mild immunodeficiency	Engineering these mutations into T cells or using T cells from individuals harboring these mutations could be an approach to replace the need to knock out endogenous TCRs for allo-ACT strategies	(51)
**CD3D**	c.202C>T	premature stop codon at residue 68	Selective lack of α/β T cells but preservation of the γ/δ T cell pool		Identify varied necessity of CD3D in α/β vs *γ*/δ T cell development.	(52, 56–58)
**CD3D**	G>A at position +5 of intron 2		Selective lack of α/β T cells but preservation of the γ/δ T cell pool			(57, 58)
**CD3G**	Haploinsufficiency		Effects γ/δ T cell pool			(59)
**CD3Z**	SNPs and splice variants in 3’UTR, intron 1 (eg rs858554), exon 7			Associated with SLE, RA, and ITP		(48, 60–64)
**CD3Z**	Hypermethylation			Associated with severe SLE phenotypes; correlates with reduced CD3ζ	Use as clinical marker to predict efficacy of checkpoint, CAR, TIL, or TCR-engineered therapies or potential for developing immunotherapy-mediated autoimmunity	(60)
**CD3G**	c.205A>T of exon 3	Premature stop codon at residue 69 of CD3γ	Decreased levels of TCR/CD3 expression	Range of disease from asymptomatic immunodeficiency to severe and fatal SCID	Reduced efficacy of TIL and checkpoint-based therapies	(69)
**CD3G**	Mutation at position -1 of exon 3	Premature stop codon	Disrupted CD3γ expression	Various autoimmune diseases		(70)
**CD3G**	c.1A>G and c.80G>C	Enrichment of hydrophobic residues at positions 6 and 7 of CDR3 chain	Decreased Treg function; impairment of CD8 T cell development only	Predisposed to autoimmunity	Increased potential for enhanced antitumor efficacy of immunotherapies but also increased risk of developing autoimmunity	(71)
CD3: ITAMS*^	Selective mutagenesis of CD3 ITAMS		Linear relationship between cumulative number of ITAM motifs and T cell proliferation rates	Mouse model with increased propensity for autoimmune disease	Precise regulation of engineered T cell activation	(73)
**CD3G**	Insertion/deletion in CD3γ promoter (rs66465034)			Increased HCC incidence	Clinical marker for disease, use as marker to predict efficacy of immunotherapies or potential for developing immunotherapy-mediated autoimmunity	(74)
**CD3D**	rs3181259			Increased recurrence in NSCLC	Clinical marker for disease, use as marker to predict efficacy of immunotherapies or potential for developing immunotherapy-mediated autoimmunity	(75)
**CD3E**	rs967591		Correlated with lower CD3ϵ expression	Shorter survival in NSCLC	Clinical marker for disease, use as marker to predict efficacy of immunotherapies or potential for developing immunotherapy-mediated autoimmunity	(76)
CD3Z: ITAMS*^		Selective mutation of one or two ITAMS of CD3ζ in CD19-CD28-CD3ζ CAR		Induction of long-term remission in pre-B acute lymphoblastic leukemia mouse model	Improved CAR or TCR-engineered therapies	(83)
**CD3Z^**		CAR with FcR*γ* instead of CD3ζ intracellular domain		Greater anti-tumor efficacy with CDζ	Improved CAR therapies	(85, 86)
**CD3Z**	rs2949655			Reduced cytotoxicity in response to BiTE treatment	Clinical marker of response to treatment or potential development of autoimmunity	(92)
**CD8A**	p.G111S		Complete lack of CD8 T cells	Recurrent infections		(103)
CD4/CD8*^	Missense, non-functional mutations - numerous		Lack of CD4 or CD8 T cell subtypes		Would not respond to checkpoint therapies and would be excluded from autologous ACT strategies	(104, 105)
CD8*^	Decreased sialylation of CD8 stalk region		Increased MHC-I binding affinity		Potential use to increase CD8 T cell activation	(123)
**CD4^**	p.Q40Y and p.T45W		Increased MHC-II binding affinity		Potential use to increase CD4 T cell activation	(101)
CD8A*^	Fused CD8α to MyD88		Enhanced CD8 T cell function		Improve ACT approaches	(120)
LCK*	Genetic knockout	LCK deficiency	Impaired T cell development, impaired activation induced T cell death		Patients would not benefit from T cell based immunotherapies	(129–132, 149)
**LCK**	c.1022T>C	Reduced CD4 and CD8 expression	Impaired TCR activation, decreased Treg levels and expansion of an oligoclonal γ/δ T cell population	Severe recurrent infections, autoimmune manifestations and panniculitis;		(138)
**LCK**	c.188-2A>G	Splice variant	Impaired LCK function and immune dysregulation	Epidermodysplasia verruciformis, recurrent bacterial infections	Patients would not benefit from T cell based immunotherapies	(139)
**LCK**	Loss of exon 7		Impaired LCK function and immune dysregulation	CVID, SCID	Patients would not benefit from T cell based immunotherapies	(140, 141)
LCK*	Deletion of C-terminal regulatory tyrosine	Truncated Lck lacking Tyr 505	Increase LCK activity, sustained T cell activation, increase IL-2 production	Sustained oncogenesis in thymoma cell line Possibility of expedited T cell exhaustion due to increased TCR and cytokine signaling	Target for therapy in T cell malignancies Engineering this mutation into tumor-reactive CAR- or TCR-engineered T cells could improve antitumor responses.	(142)
**LCK**	t(1;7)(p34;q34)	Chromosomal translocation	Increased LCK activity that is correlated with breakpoint location and disease severity	T cell acute lymphoblastic leukemia	Target for therapy in T cell malignancies Engineering this translocation into tumor-reactive TCR-, CAR- -TCR-engineered T cells or TILs could improve antitumor responses.	(144)
**LCK binding partner^**	Mutation of PYAP motif within CD28	Deleted LCK-binding motif in CD28 (ΔCD28) in CARs	Reduced IL-2 production, improved tumor control	Stable disease with pleural mesothelioma	Enhancement of CAR T cell efficacy	(145–147)
**LCK**	rs10914542		Impairs TCR activation and proliferation	Increase risk of T1D	Predict efficacy of ICI therapy	(151)
**LCK**	p.G85W of exon 4			Associated with Sjogren syndrome, SLE, RA	Possible increased risk of developing autoimmune complications	(152)
FYN*^	Genetic knockout	Loss of Fyn expression	Promotes differentiation of CD4+ T cells towards Treg (away from Th17)		Activating FYN could promote Th17 phenotype	(163)
**ZAP70**	Mutations in SH2 and kinase domains: c.169G>A, c.448C>T, c.1602C>T, c.1603G>A, c.1729C>T, c.1763C>A, c.1833G>A, c.1923A>T	Loss or destabilization of protein transcript	Complete lack of functional T cells	Severe immunodeficiency	Patients would not benefit from T cell based immunotherapies	(175–182)
**ZAP70**	Polymorphisms in coding region (rs17695937, rs13420683) or 3’UTR (rs2278699)			Associated with psoriasis, T1DM, IBD, RA		(183–186)
**ZAP70**	Intron variant (rs7425883)		Aberrant expression of ZAP70 and enhanced BCR signaling in B cell CLL	Associated with decreased risk of developing non-Hodgkin lymphoma	ZAP70 as prognostic marker of disease Clinical marker for disease, use as marker to predict efficacy of immunotherapies or potential for developing immunotherapy-mediated autoimmunity	(187)
ZAP70*^	Point mutations in second SH2 interdomain, or paired mutations in kinase region		Aberrant thymic selection permissive to higher affinity self-reactive clones		Engineering this mutation into CAR- or TCR-engineered T cells could improve antitumor responses.	(189–191)
**ZAP70^**	p.Y292F, p.Y315F, p.Y492F	Lack of phosphorylation at key inhibitory residues	De-inhibited ZAP70 signaling	Increased T cell activation	Could be used to enhance T cell activation for immunotherapy	(192)
**ZAP70^**	p.M414A, p.M414A/p.C405V	Selective inhibition of engineered ZAP70 with PPI-derived inhibitor	Impaired catalytic activity (not observed in Tregs)	Can selectively turn “off” ZAP70 in engineered T cells	Similar approaches could be used to turn off adoptively transferred T cells	(193, 194)
**ZAP70^**	Tetracycline-inducible ZAP70 promoter	Selectively turn on ZAP70 transcription			Selective over-expression and activation of T cells	(195)
**ZAP70^**	Fusion of analog-sensitive ZAP70 to the ligand binding domain of the estrogen receptor	Selectively turn on and off ZAP70	Turn on and off ZAP70 in minute-by-minute timeframe		Tight real-time control of engineered T cell activation states	(196)
**LAT^**	p.Y132F	Defective binding with PLC*γ*-1			Could be used to fine-tune T cell activation states	(204)
**LAT^**	p.Y110F and p.Y226F	Required for ERK activation			Could be used to fine-tune T cell activation states	(204)
**LAT^**	p.Y171F	Essential for PI3K activation			Could be used to fine-tune T cell activation states	(205)
**LAT^**	p.Y171F p.Y191F	Required for Gads binding			Could be used to fine-tune T cell activation states	(206)
**LAT^**	p.Y171F, p.Y191F, p.Y225F	Required for Grb2 binding			Could be used to fine-tune T cell activation states	(206)
**LAT^**	p.S38A, p.S40A, p.S106A, p.S164A, p.S108A	Decreased PLC*γ*-1 and SLP-76 binding, increased ZAP70 phosphorylation	Reduced IL-2 production		Could be used to fine-tune T cell activation states	(207)
LAT*^	Mutation of distal 4 Tyr residues to Phe		Mimics LAT-/- in mice; T cell development blocked at DN3 stage		Patients likely to demonstrate weakened responses to T cell based immunotherapies	(209)
LAT*^	p.Y136F	Impaired PLCγ binding	MHC-independent constitutive TH2 activation; T cell dependent B cell hyperproliferation and antibody production	Systemic autoimmune disease	Increase T cell activation	(215)
LAT*^	p.Y136F		Disrupted thymic positive and negative selection			(216)
LAT*^	p.Y136F	Blocked PLCγ dependent functions, ERK signaling maintained	Impaired T cell development	Lymphoproliferative disorder	Increase T cell proliferation	(217)
LAT*^	c.268_269del	Loss of the cytoplasmic tail of LAT and phosphorylation sites	Decreased circulating T cells, aberrant ERK signaling	immunodeficiency with simultaneous autoimmunity	Patients likely to demonstrate weakened responses to T cell based immunotherapies	(222)
**LAT^**	p.K52R and p.K204R	Decreased ubiquitylation	Enhanced T cell signaling		Enhance T cell activation	(224)
**LAT^**	p.G131D or p.G131E	Increased phosphorylation kinetics of Y132, increased speed and magnitude of PLCγ activation	Increased sensitivity of T cells to weak antigen stimulation		Enhance T cell activation	(225)
**ITK**	Gain of function (t(5;9)(q33;q22))			T cell lymphomas	Target for therapy	(227)
**ITK**	Two patients: 1) p.R29H; 2) p.D500T, p.F501L, and p.M503X	ITK loss of function	Loss of T cell regulation	Lymphoproliferative diseases	Increase expansion of engineered T cells	(228)
**ITK**	p.Q17X	ITK deficiency		Idiopathic CD4+ T cell lymphopenia		(229)
**ITK**	c.196C>T in promoter region	Increased ITK transcription		Associated with asthma	Could be used to lower the threshold for T cell activation	(230)
**FYB**	rs6863066 rs358501		Low levels of LAT-PLCγ-1 interactions; skewing towards aberrant Th2 phenotype	Susceptibility to asthma	Patients likely to demonstrate weakened responses to T cell based immunotherapies	(231)
**VAV1^**	Deletion of nucleotides 1-67	Creates “oncogenic VAV1”	Decreased calcium mobilization	Linked to oncogenesis	Patients likely to demonstrate suboptimal T cell responses to immunotherapies	(236)
**VAV1**	p.E59K, p.D517E	Constitutively active or highly stable and overexpressed VAV1	Oncogenesis	Driver mutations in human lung adenocarcinoma	Potential target for therapy	(237)
ADAP*^		ADAP KO in adoptively transferred T cells	Reduced PD-1 expression, increased anti-tumor efficacy		Improve adoptive T cell therapy	(240)

Detailed tabulation of all major genetic and molecular changes discussed in this review, with the clinical or phenotypic outcomes and potential relevance to immunotherapy as indicated. Of note, this is not an exhaustive list. For additional detailed inquiries, please refer to the references cited in the text.

*studies in mice only.

^mutational studies or engineered mutations.

AD, Atopic dermatitis; CVID, Common Variable Immune Deficiency; HCC, Hepatocellular Carcinoma; SLE, Systemic Lupus Erythematosus; IBD, Inflammatory Bowel Disease; CD, Crohn’s disease; ICB/ICI, Immune Checkpoint Blockade/Inhibitors; T1D, Type 1 Diabetes; X-SCID/SCID, X-Linked Severe Combined Immunodeficiency; PSC, primary sclerosing cholangitis; MSMD, Mendelian Susceptibility to Mycobacterial Disease; MS, Multiple Sclerosis; ITP, Immune Thrombocytopenia; UC, Ulcerative Colitis; RA, Rheumatoid Arthritis; TB, Tubercolosis.

## Author Contributions

AK and NL wrote the manuscript and prepared the figures. AC prepared the table and provided editing and input into the manuscript. ED oversaw the entire project providing guidance as well as detailed review and editing of all aspects of the paper. All authors contributed to the article and approved the submitted version.

## Funding

VA Merit Award I01BX004935; NCI R01 CA207913; NCI T32 CA23673402; University of Colorado Comprehensive Cancer Center

## Conflict of Interest

The authors declare that the research was conducted in the absence of any commercial or financial relationships that could be construed as a potential conflict of interest.
